# Supplementary material for: Achieving low-power single-wavelength-pair nanoscopy with NIR-II continuous-wave laser for multi-chromatic probes
Source: Nat Commun. 2022 May 23;13:2843. doi: 10.1038/s41467-022-30114-z (PMC9126916; doi:10.1038/s41467-022-30114-z)
Supplement: Supplementary file 1 — Supplementary Information [file 41467_2022_30114_MOESM1_ESM.pdf]

Supplementary Information for

Achieving low-power single-wavelength-pair nanoscopy with NIR-II continuous-wave laser for multi-chromatic probes

Xin Guo[1]†, Rui Pu[1]†, Zhimin Zhu[1], Shuqian Qiao[1], Yusen Liang[1], Bingru Huang[1], Haichun Liu[2], Lucía Labrador-Páez[2], Uliana Kostiv[2], Pu Zhao[1], Qiusheng Wu[1], Jerker Widengren[2] & Qiuqiang Zhan[1,3*]

[1]Centre for Optical and Electromagnetic Research, Guangdong Provincial Key Laboratory of Optical Information Materials and Technology, National Center for International Research on Green Optoelectronics, South China Academy of Advanced Optoelectronics, South China Normal University, Guangzhou 510006, P. R. China.

[2]Experimental Biomolecular Physics, Department of Applied Physics, KTH Royal Institute of Technology, SE-106 91, Stockholm, Sweden.

[3]MOE Key Laboratory and Guangdong Provincial Key Laboratory of Laser Life Science, College of Biophotonics, South China Normal University, Guangzhou 510631, P. R. China.

†These authors contributed equally: Xin Guo, Rui Pu.

*Corresponding author, ✉email: zhanqiuqiang@m.scnu.edu.cn.

This PDF file includes:

Supplementary Methods: materials and agents
Supplementary Notes 1 to 2
Supplementary Figures 1 to 17
Supplementary Tables 1 to 6
Supplementary References

Table of Contents

Supplementary Methods: Materials and agents……………………..……….………………………...…3

Supplementary Note 1 STExD simulation for $NaYF_4$:Nd/Yb/Er system…………………………...…4

Supplementary Note 2 STExD simulation for $NaYF_4$:Nd/Yb/Ho system……………….……………6

Supplementary Fig. 1 Simulated population inversion between $^4F_{3/2}$ and $^4I_{11/2}$ in Nd^{3+}……………..……..8

Supplementary Fig. 2 Schematic diagram of the optical setup…………………..………….……….…9

Supplementary Fig. 3 TEM images of the as-prepared nanoparticles……………………..……………10

Supplementary Fig. 4 EDS and ICP analysis of the as-prepared nanoparticles……………….…...….11

Supplementary Fig. 5 Nd^{3+} absorption spectrum and emission inhibition under 800 nm excitation….12

Supplementary Fig. 6 Emission intensities of $NaYF_4$:Nd nanoparticles versus excitation intensity….13

Supplementary Fig. 7 Energy diagram of cascade amplified depletion modelling in STExD………...14

Supplementary Fig. 8 Energy diagram for numerical simulation in Nd system……….…………...15

Supplementary Fig. 9 Simulated emission intensities of $NaYF_4$:Nd nanoparticles versus depletion intensity……………………………………………………………………………16

Supplementary Fig. 10 Emission spectra and intensities versus Nd^{3+} doping concentration……..…...17

Supplementary Fig. 11 Time-resolved kinetics of the $^2P_{1/2}$ state in Nd-nanoparticles…………….…18

Supplementary Fig. 12 Emission intensities of $NaYF_4$:Nd/Yb/Er(Ho) nanoparticles versus excitation intensity……………………………………………………………………….…19

Supplementary Fig. 13 Energy diagrams for numerical simulation in Nd/Yb/Er(Ho) system…..…...20

Supplementary Fig. 14 Simulated emission intensities of $NaYF_4$:Nd/Yb/Er(Ho) nanoparticles versus depletion intensity…………………….…………...……………………21

Supplementary Fig. 15 Schematic energy diagrams for STExD mechanism in other emitters.……..…22

Supplementary Fig. 16 The emission spectra of nanoprobes for two-color STExD imaging……….…23

Supplementary Fig. 17 TEM image and spectrum of phalloidin-$NaYF_4$:Nd nanoparticles……...……24

Supplementary Table 1 Comparison of the theoretical saturation intensity of different emitters…..…25

Supplementary Table 2 The list of the as-synthesized samples studied in this work……...…..…….26

Supplementary Table 3 Measured depletion efficiencies of Nd-sensitizing nanoparticles..………….27

Supplementary Table 4 The parameters used in the simulation for Nd system…….………………..28

Supplementary Table 5 The parameters used in the simulation for Nd/Yb/Er system………………...29

Supplementary Table 6 The parameters used in the simulation for Nd/Yb/Ho system…….…..……..30

Supplementary References…………………….…………………………………………...31

Supplementary Methods: Materials and agents

Neodymium (III) acetate hydrate (99.9%), yttrium (III) acetate hydrate (99.9%), gadolinium (III) acetate hydrate (99.9%), ytterbium (III) acetate hydrate (99.9%), erbium (III) acetate hydrate (99.9%), thulium (III) acetate hydrate (99.9%), holmium (III) acetate hydrate (99.9%), praseodymium (III) acetate hydrate (99.9%), cerium (III) acetate hydrate (99.9%), terbium (III) acetate hydrate (99.9%), europium (III) acetate hydrate (99.9%), N-(3-Dimethylaminopropyl)-N'-ethylcarbodiimide hydrochloride (EDC, 98%), Poly (acrylic acid) (PAA, sodium salt, Mw~1200) were purchased from Sigma-Aldrich. Sodium hydroxide (NaOH, 99%), ammonium fluoride (NH_4F, ≥99.99%), 1-octadecene (ODE, ≥ 90%(GC)), oleic acid (OA, AR), Nitrosyl tetrafluoroborate ($NOBF_4$, 95%), N, N-Dimethylformamide (DMF, 99.5%), sodium oleate (AR, 99.5%), nitric acid (65-68%) and cyclohexane (AR, 99.5%) were purchased from Aladdin®, China. 2-(N-Morpholino) ethanesulfonic (MES) acid Buffer (0.1 M, pH = 6.0) were purchased from Leagene Biotechnology co., Beijing. Methanol (AR), ethanol (AR) and dimethylsulfoxide (DMSO, AR) were purchased from Sinopharm Chemical Reagent Co., China. N-Hydroxysuccinimide (NHS, 98%), Phosphate-buffered Saline (PBS) and ProLong™ Gold Antifade Mountant were purchased from Thermo Fisher Scientific. Amino-Phalloidin was purchased from Shanghai Maokang Biotechnology Co. Ltd., China. 2-[4-(2-hydroxyethyl) piperazin-1-yl] ethanesulfonic acid (HEPES) buffer (0.01 M, pH = 7.3), immunostaining fixative, immunostaining permeate and immunostaining blocking solution were purchased from Beyotime Biotechnology. Hela cell line was purchased from Procell, Wuhan. All the reagents were used as received without further purification.

Supplementary Notes 1: STExD simulation for $NaYF_4$:Nd/Yb/Er system

The rate equations of the proposed STExD process in $NaYF_4$:Nd/Yb/Er (3/1/5%) nanoparticles (shown in Supplementary Fig. 13a) are derived as follows:

$Nd^{3+}(^4I_{9/2})$: $$\frac{dn_{\mathrm{Nd0}}}{dt} = -\rho_1\sigma_{\mathrm{Nd0}}n_{\mathrm{Nd0}} + w_{\mathrm{Nd}}n_{\mathrm{Nd2}}n_{\mathrm{Yb0}} + \beta_{\mathrm{Nd1}}n_{\mathrm{Nd1}} \quad (1)$$

$Nd^{3+}(^4I_{11/2})$: $$\frac{dn_{\mathrm{Nd1}}}{dt} = -\rho_2\sigma_{\mathrm{Nd12}}n_{\mathrm{Nd1}} + \rho_2\sigma_{\mathrm{Nd21}}n_{\mathrm{Nd2}} - \beta_{\mathrm{Nd1}}n_{\mathrm{Nd1}} \quad (2)$$

$Nd^{3+}(^4F_{3/2})$: $$\frac{dn_{\mathrm{Nd2}}}{dt} = \rho_2\sigma_{\mathrm{Nd12}}n_{\mathrm{Nd1}} - \rho_2\sigma_{\mathrm{Nd21}}n_{\mathrm{Nd2}} - w_{\mathrm{Nd}}n_{\mathrm{Nd2}}n_{\mathrm{Yb0}} + \beta_{\mathrm{Nd3}}n_{\mathrm{Nd3}} \quad (3)$$

$Nd^{3+}(^4F_{7/2})$: $$\frac{dn_{\mathrm{Nd3}}}{dt} = \rho_1\sigma_{\mathrm{Nd0}}n_{\mathrm{Nd0}} - \beta_{\mathrm{Nd3}}n_{\mathrm{Nd3}} \quad (4)$$

$Yb^{3+}(^2F_{7/2})$: $$\frac{dn_{\mathrm{Yb0}}}{dt} = -w_{\mathrm{Nd}}n_{\mathrm{Nd2}}n_{\mathrm{Yb0}} + w_1n_{\mathrm{Yb1}}n_0 + w_2n_{\mathrm{Yb1}}n_1 + w_3n_{\mathrm{Yb1}}n_2 + w_4n_{\mathrm{Yb1}}n_3 + w_5n_{\mathrm{Yb1}}n_4 \quad (5)$$

$Yb^{3+}(^2F_{5/2})$: $$\frac{dn_{\mathrm{Yb1}}}{dt} = w_{\mathrm{Nd}}n_{\mathrm{Nd2}}n_{\mathrm{Yb0}} - w_1n_{\mathrm{Yb1}}n_0 - w_2n_{\mathrm{Yb1}}n_1 - w_3n_{\mathrm{Yb1}}n_2 - w_4n_{\mathrm{Yb1}}n_3 - w_5n_{\mathrm{Yb1}}n_4 \quad (6)$$

$Er^{3+}(^4I_{15/2})$: $$\frac{dn_0}{dt} = -w_1n_{\mathrm{Yb1}}n_0 + \beta_1n_1 + \frac{n_1}{\tau_1} + \frac{n_3}{\tau_3} + \frac{n_4}{\tau_4} + \frac{n_6}{\tau_6} \quad (7)$$

$Er^{3+}(^4I_{13/2})$: $$\frac{dn_1}{dt} = -w_2n_{\mathrm{Yb1}}n_1 - \beta_1n_1 + \beta_2n_2 - \frac{n_1}{\tau_1} + \frac{n_7}{\tau_7} + \frac{n_8}{\tau_8} \quad (8)$$

$Er^{3+}(^4I_{11/2})$: $$\frac{dn_2}{dt} = w_1n_{\mathrm{Yb1}}n_0 - w_3n_{\mathrm{Yb1}}n_2 - cn_2n_5 - \beta_2n_2 + \beta_3n_3 \quad (9)$$

$Er^{3+}(^4F_{9/2})$: $$\frac{dn_3}{dt} = -\rho_2\sigma_{36}n_3 + w_2n_{\mathrm{Yb1}}n_1 - w_4n_{\mathrm{Yb1}}n_3 + 2cn_2n_5 - \beta_3n_3 + \beta_4n_4 - \frac{n_3}{\tau_3} \quad (10)$$

$Er^{3+}(^4S_{3/2})$: $$\frac{dn_4}{dt} = -\rho_2\sigma_{48}n_4 - w_5n_{\mathrm{Yb1}}n_4 - \beta_4n_4 + \beta_5n_5 - \frac{n_4}{\tau_4} \quad (11)$$

$Er^{3+}(^4F_{7/2})$: $$\frac{dn_5}{dt} = +w_3n_{\mathrm{Yb1}}n_2 - cn_2n_5 - \beta_5n_5 + \beta_6n_6 \quad (12)$$

$Er^{3+}(^2H_{9/2})$: $$\frac{dn_6}{dt} = \rho_2\sigma_{36}n_3 + w_4n_{\mathrm{Yb1}}n_3 - \beta_6n_6 + \beta_7n_7 - \frac{n_6}{\tau_6} \quad (13)$$

$Er^{3+}(^4G_{11/2})$: $$\frac{dn_7}{dt} = -\beta_7n_7 + \beta_8n_8 - \frac{n_7}{\tau_7} \quad (14)$$

$Er^{3+}(^2K_{15/2})$: $$\frac{dn_8}{dt} = \rho_2\sigma_{48}n_4 + w_5n_{\mathrm{Yb1}}n_4 - \beta_8n_8 - \frac{n_8}{\tau_8} \quad (15)$$

Here $n_{\mathrm{Nd}i}$ (i = 0 to 3) represents the population of Nd^{3+} ions on the $^4I_{9/2}$, $^4I_{11/2}$, $^4F_{3/2}$, $^4F_{7/2}$ states, respectively. $n_{\mathrm{Yb}i}$ (i = 0, 1) represents the population of Yb^{3+} ions on the $^2F_{7/2}$ and $^2F_{5/2}$ states, respectively. n_i (i = 0 to 8) represents the population of Er^{3+} ions on the $^4I_{15/2}$, $^4I_{13/2}$, $^4I_{11/2}$, $^4F_{9/2}$, $^4S_{3/2}$, $^4F_{7/2}$, $^2H_{9/2}$, $^4G_{11/2}$ and $^2K_{15/2}$ states, respectively. τ_i (i = 3, 4, 6, 7, 8) represents the radiative lifetimes of the $^4F_{9/2}$, $^4S_{3/2}$, $^2H_{9/2}$, $^4G_{11/2}$ and $^2K_{15/2}$ states of Er^{3+} ions, respectively. β_i (i = 1 to 8) represents the nonradiative decay rates of the

$^4I_{15/2}$, $^4I_{13/2}$, $^4I_{11/2}$, $^4F_{9/2}$, $^4S_{3/2}$, $^4F_{7/2}$, $^2H_{9/2}$, $^4G_{11/2}$ and $^2K_{15/2}$ states of Er^{3+} ions, respectively. β_{Ndi} (i = 1, 3) represents the nonradiative decay rates of the $^4I_{11/2}$ and $^4F_{7/2}$ states of Nd^{3+} ions, respectively. w_i (i = 1 to 5) denotes the energy transfer upconversion coefficients from Yb^{3+} to the $^4I_{15/2}$, $^4I_{13/2}$, $^4I_{11/2}$, $^4F_{9/2}$ and $^2K_{15/2}$ states of Er^{3+}, respectively. w_{Nd} denotes the energy transfer coefficient from the $^4F_{3/2}$ state of Nd^{3+} to the $^2F_{7/2}$ state of Yb^{3+}. c denotes the coefficient of cross-relaxation process: $^4F_{7/2} + {}^4I_{11/2} \rightarrow {}^4F_{9/2} + {}^4F_{9/2}$ in Er^{3+}. σ_{Nd0}, σ_{Nd12} and σ_{Nd21} denote the absorption cross-section for the ground state absorption process: $^4I_{9/2} \rightarrow {}^4F_{7/2}$, the absorption cross-section for the excited state absorption process: $^4I_{11/2} \rightarrow {}^4F_{3/2}$, and the stimulated emission cross-section for the stimulated emission process: $^4F_{3/2} \rightarrow {}^4I_{11/2}$ in Nd^{3+}, respectively. σ_{36} and σ_{48} denotes the absorption cross-section for the excited state absorption process: $^4F_{9/2} \rightarrow {}^2H_{9/2}$ and $^4S_{3/2} \rightarrow {}^2K_{15/2}$ in Er^{3+}. ρ_1 and ρ_2 are the laser intensities of the 740 nm and 1064 nm beams. The values used for the main parameters are tabulated in Supplementary Table 5. Similar to the simulation of the $NaYF_4$: Nd system, increasing the depletion intensity cane consume the population of $^4F_{9/2}$ and $^4S_{3/2}$ states reflect the depletion performance of the 655 nm and 540 nm emission bands. The related simulation results were shown in Supplementary Fig. 14a.

Supplementary Notes 2: STExD simulation for $NaYF_4$:Nd/Yb/Ho system

The rate equations of the proposed STExD process of $NaYF_4$:Nd/Yb/Ho (3/0.5/2%) nanoparticles (shown in Supplementary Fig. 13b) are derived as follows:

$Nd^{3+}(^4I_{9/2})$: $\frac{dn_{\mathrm{Nd0}}}{dt} = -\rho_1\sigma_{\mathrm{Nd0}}n_{\mathrm{Nd0}} + \beta_{\mathrm{Nd1}}n_{\mathrm{Nd1}} + w_{\mathrm{Nd}}n_{\mathrm{Nd2}}n_{\mathrm{Yb0}}$ (16)

$Nd^{3+}(^4I_{11/2})$: $\frac{dn_{\mathrm{Nd1}}}{dt} = -\rho_2\sigma_{\mathrm{Nd12}}n_{\mathrm{Nd1}} + \rho_2\sigma_{\mathrm{Nd21}}n_{\mathrm{Nd2}} - \beta_{\mathrm{Nd1}}n_{\mathrm{Nd1}}$ (17)

$Nd^{3+}(^4F_{3/2})$: $\frac{dn_{\mathrm{Nd2}}}{dt} = \rho_2\sigma_{\mathrm{Nd12}}n_{\mathrm{Nd1}} - \rho_2\sigma_{21}n_{\mathrm{Nd2}} - w_{\mathrm{Nd}}n_{\mathrm{Nd2}}n_{\mathrm{Yb0}} + \beta_{\mathrm{Nd3}}n_{\mathrm{Nd3}}$ (18)

$Nd^{3+}(^4F_{7/2})$: $\frac{dn_{\mathrm{Nd3}}}{dt} = \rho_1\sigma_{\mathrm{Nd0}}n_{\mathrm{Nd0}} - \beta_{\mathrm{Nd3}}n_{\mathrm{Nd3}}$ (19)

$Yb^{3+}(^2F_{7/2})$: $\frac{dn_{\mathrm{Yb0}}}{dt} = -w_{\mathrm{Nd}}n_{\mathrm{Nd2}}n_{\mathrm{Yb0}} + w_1n_{\mathrm{Yb1}}n_0 + w_2n_{\mathrm{Yb1}}n_1 + w_3n_{\mathrm{Yb1}}n_2 + w_4n_{\mathrm{Yb1}}n_3$ (20)

$Yb^{3+}(^2F_{5/2})$: $\frac{dn_{\mathrm{Yb1}}}{dt} = w_{\mathrm{Nd}}n_{\mathrm{Nd2}}n_{\mathrm{Yb0}} - w_1n_{\mathrm{Yb1}}n_0 - w_2n_{\mathrm{Yb1}}n_1 - w_3n_{\mathrm{Yb1}}n_2 - w_4n_{\mathrm{Yb1}}n_3$ (21)

$Ho^{3+}(^5I_8)$: $\frac{dn_0}{dt} = -w_1n_{\mathrm{Yb1}}n_0 - cn_0n_6 + \beta_1n_1 + \frac{n_2}{\tau_2} + \frac{n_5}{\tau_5} + \frac{n_6}{\tau_6} + \frac{n_7}{\tau_7}$ (22)

$Ho^{3+}(^5I_7)$: $\frac{dn_1}{dt} = -\rho_1\sigma_{16}n_1 - w_2n_{\mathrm{Yb1}}n_1 + cn_0n_6 - \beta_1n_1 + \beta_2n_2$ (23)

$Ho^{3+}(^5I_6)$: $\frac{dn_2}{dt} = -\rho_2\sigma_{26}n_2 + w_1n_{\mathrm{Yb1}}n_0 - w_3n_{\mathrm{Yb1}}n_2 - \beta_2n_2 + \beta_3n_3 - \frac{n_2}{\tau_2}$ (24)

$Ho^{3+}(^5I_5)$: $\frac{dn_3}{dt} = -\rho_2\sigma_{37}n_3 - w_4n_{\mathrm{Yb1}}n_3 - \beta_3n_3 + \beta_4n_4$ (25)

$Ho^{3+}(^5I_4)$: $\frac{dn_4}{dt} = cn_0n_6 - \beta_4n_4 + \beta_5n_5$ (26)

$Ho^{3+}(^5F_5)$: $\frac{dn_5}{dt} = w_2n_{\mathrm{Yb1}}n_1 - \beta_5n_5 + \beta_6n_6 - \frac{n_5}{\tau_5}$ (27)

$Ho^{3+}(^5F_4)$: $\frac{dn_6}{dt} = \rho_1\sigma_{16}n_1 + \rho_2\sigma_{26}n_2 + w_3n_{\mathrm{Yb1}}n_2 - cn_0n_6 - \beta_6n_6 + \beta_7n_7 - \frac{n_6}{\tau_6}$ (28)

$Ho^{3+}(^5F_2)$: $\frac{dn_7}{dt} = \rho_2\sigma_{37}n_3 + w_4n_{\mathrm{Yb1}}n_3 - \beta_7n_7 - \frac{n_7}{\tau_7}$ (29)

Here $n_{\mathrm{Nd}i}$ (i = 0 to 3) represents the population of Nd^{3+} ions on the $^4I_{9/2}$, $^4I_{11/2}$, $^4F_{3/2}$, $^4F_{7/2}$ states, respectively. $n_{\mathrm{Yb}i}$ (i = 0, 1) represents the population of Yb^{3+} ions on the $^2F_{7/2}$ and $^2F_{5/2}$ states, respectively. n_i (i = 0 to 7) represents the population densities of Ho^{3+} ions on the $^5I_8$, $^5I_7$, $^5I_6$, $^5I_5$, $^5I_4$, $^5F_5$, $^5F_4$ and $^5F_2$ states, respectively. τ_i (i = 5, 6, 7) represents the radiative lifetimes of the $^5F_5$, $^5F_4$ and $^5F_2$ states of Ho^{3+} ions, respectively. β_i (i = 1 to 7) represents the nonradiative decay rates of the $^5I_7$, $^5I_6$, $^5I_5$, $^5I_4$, $^5F_5$, $^5F_4$ and $^5F_2$ states of Ho^{3+} ions, respectively. $\beta_{\mathrm{Nd}i}$ (i = 1, 3) represents the nonradiative decay rates of the $^4I_{11/2}$ and $^4F_{7/2}$ states of Nd^{3+} ions, respectively. w_i (i = 1 to 4) denotes the energy transfer upconversion coefficients

from Yb^{3+} to the $^5I_8$, $^5I_7$, $^5I_6$ and $^5I_5$ states of Ho^{3+}, respectively. w_{Nd} denotes the energy transfer coefficient from the $^4F_{3/2}$ state of Nd^{3+} to the $^2F_{7/2}$ state of Yb^{3+}. c denotes the coefficient of cross relaxation process: $^5I_8 + {}^5F_4 \rightarrow {}^5I_7 + {}^5I_4$ in Ho^{3+}. σ_{Nd0}, σ_{Nd12} and σ_{Nd21} denote the absorption cross-section for the ground state absorption process: $^4I_{9/2} \rightarrow {}^4F_{7/2}$, the absorption cross-section for the excited state absorption process: $^4I_{11/2} \rightarrow {}^4F_{3/2}$, and the stimulated emission cross-section for the stimulated emission process: $^4F_{3/2} \rightarrow {}^4I_{11/2}$ in Nd^{3+}, respectively. σ_{16}, σ_{26} and σ_{37} denotes the absorption cross-section for the excited state absorption process: $^5I_7 \rightarrow {}^5F_4$, $^5I_6 \rightarrow {}^5F_4$ and $^5I_5 \rightarrow {}^5F_3$ in Ho^{3+}. ρ_1 and ρ_2 are the laser intensities of the 740 nm and 1064 nm beams. The values used for the main parameters are tabulated in Supplementary Table 6 and the calculated results are shown in Supplementary Fig. 14b.

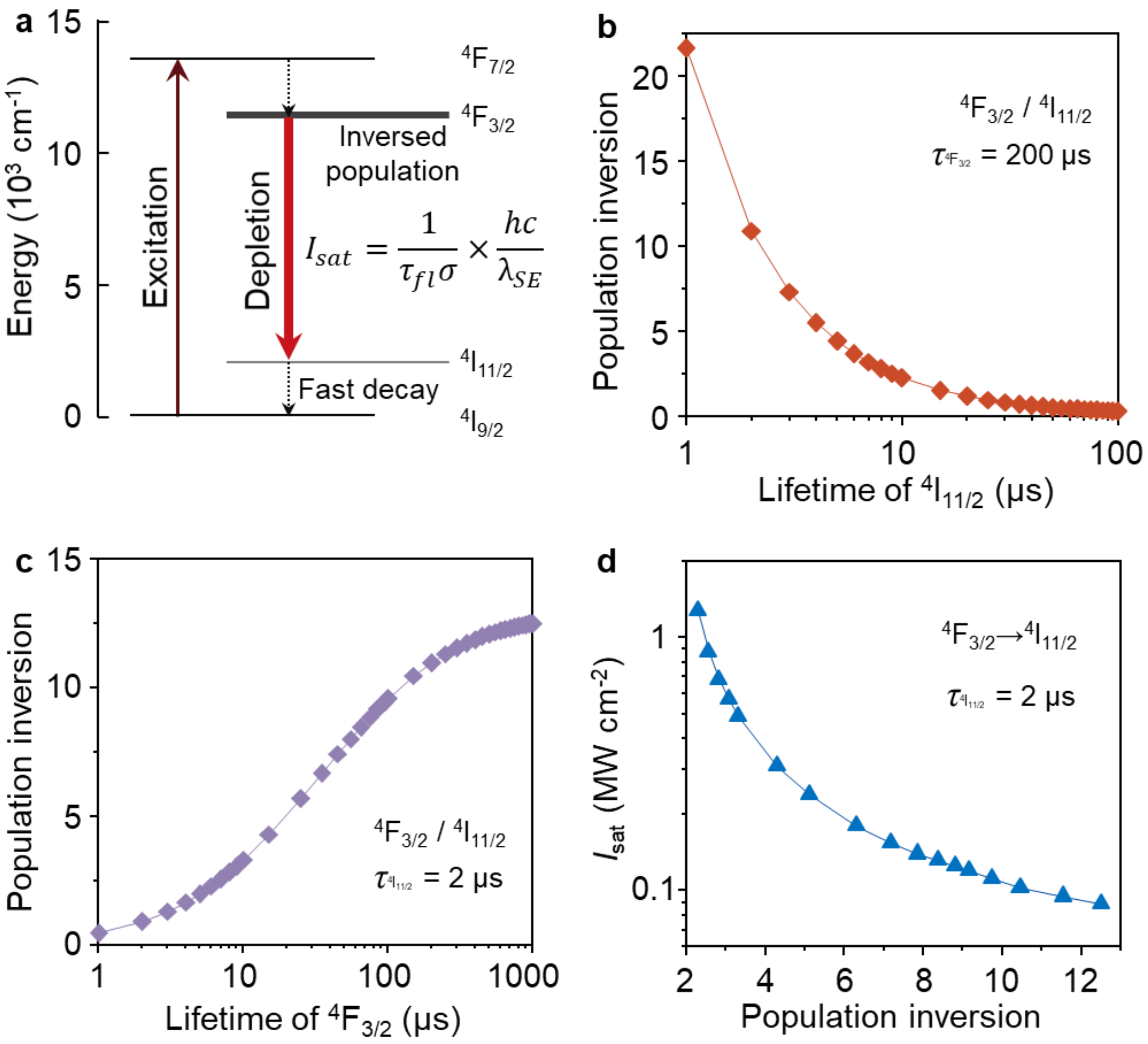

Supplementary Fig. 1 | Simulated population inversion between the $^4F_{3/2}$ and $^4I_{11/2}$ states for different decay lifetimes. a Classic four-level system in Nd^{3+} offers a large emission cross-section (σ), a long energy level lifetime (τ_{fl}) and a long depletion wavelength (λ_{SE}), allowing efficient stimulated emission depletion featuring low saturation intensity (I_{sat}). c and h denote the speed of light and Plank's constant. **b** Population inversion for different decay lifetimes of $^4I_{11/2,}$ with the $^4F_{3/2}$ state lifetime τ = 200 μs. **c** Population inversion for different decay lifetimes of $^4F_{3/2,}$ with the $^4I_{11/2}$ state lifetime τ = 2 μs. **d** Simulated I_{sat} of the one-photon emission from the $^4F_{3/2}$ state, with different population inversion ratios between $^4F_{3/2}$ and $^4I_{11/2}$, showing a significant decrease with the increasing inverted population.

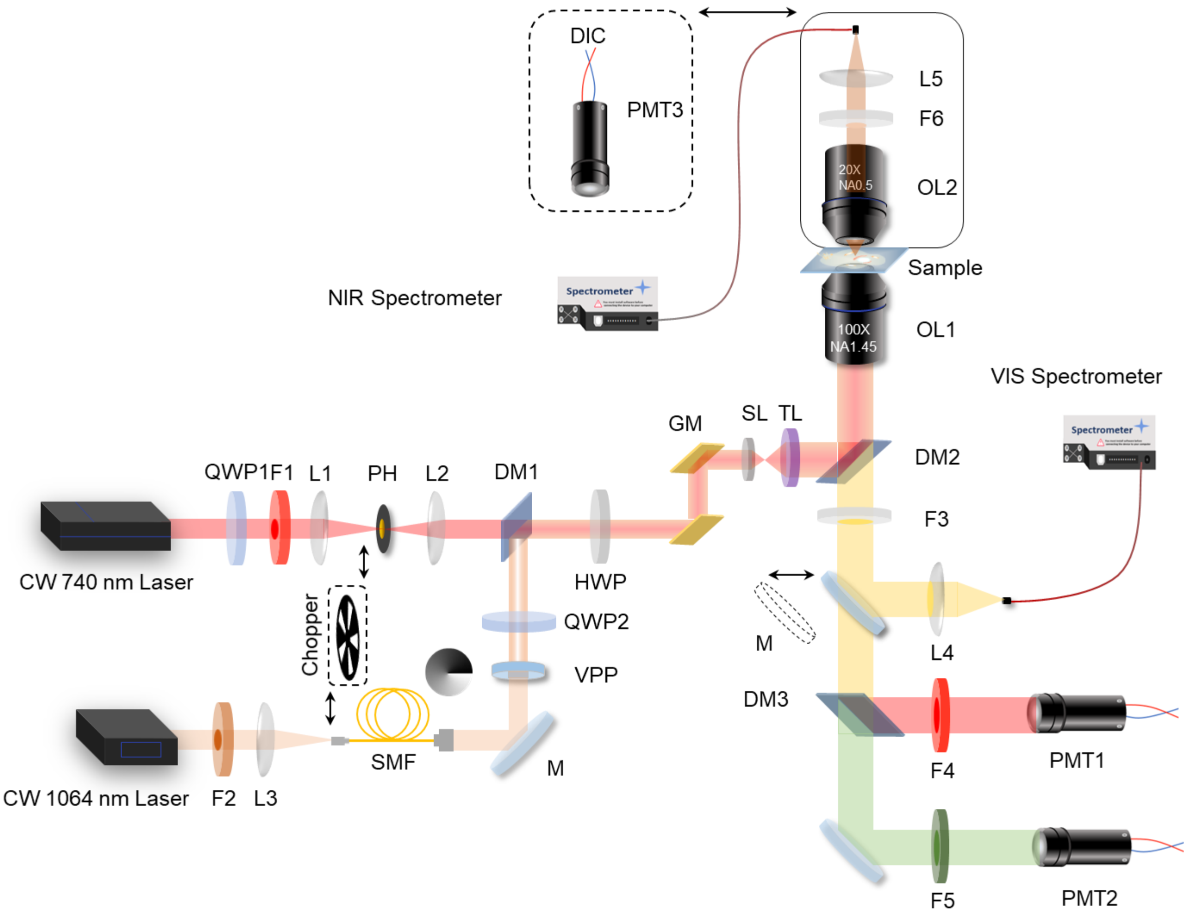

Supplementary Fig. 2 | Schematic diagram of the optical setup employed in this study. F1: 725/40 nm band-pass filter; F2: 1064/5-25 nm band-pass filter; F3: 694 nm short-pass filter; F4: 645/30 nm band-pass filter; F5: 590/33 nm band-pass filter; F6: 935/170 nm band-pass filter; HWP: half-wave plate; QWP1-QWP2: quarter-wave plates; L1-L3: 50 mm focus length lenses; L4-L5: 25 mm focus length lenses; SMF: single mode fiber; TL: tube lens; SL: scan lens; PH: 25 μm pinhole; VPP: 1064 nm vortex phase plate. DM1: 820 nm short-pass dichroic mirror; DM2: 690 nm short-pass dichroic mirror; DM3: 610 nm short-pass dichroic mirror; OL1: 100× oil immersed objective lens; OL2: 20× objective lens; GM: galvanometer scanning mirrors; M: sliver reflection mirrors; PMT1, PMT2, PMT3: photomultiplier tubes.

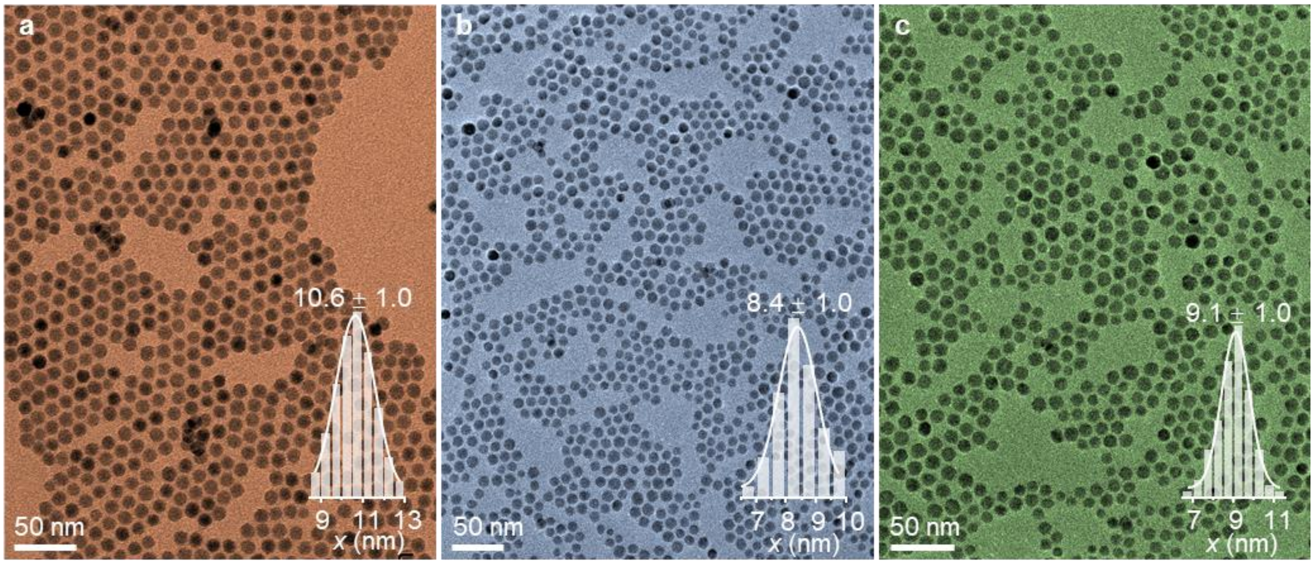

Supplementary Fig. 3 | TEM images of the (**a**) $NaYF_4$:Nd (3%, 10.6 ± 1.0 nm), (**b**) Nd/Yb/Er (3/1/5%, 8.4 ± 1.0 nm) and (**c**) Nd/Yb/Ho (3/0.5/2%, 9.1 ± 1.0 nm) nanoparticles. Scale bar: 50 nm. Each experiment was repeated 3 times independently with similar results.

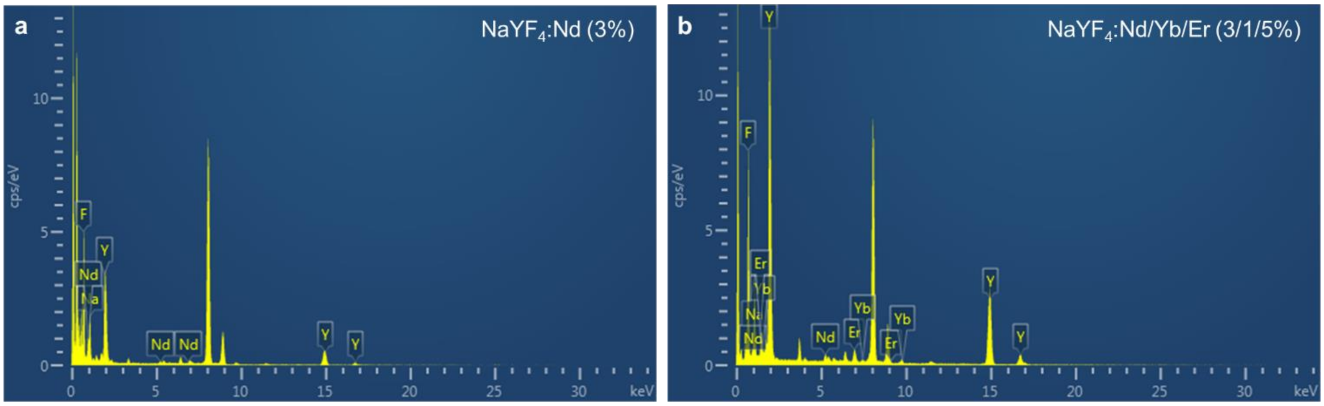

c

Sample	Dopant concentrations	ICP results
$NaYF_4$:Nd	3%	2.41 ± 0.46%
$NaYF_4$:Nd/Yb/Er	3/1/5%	2.58 ± 0.52 / 0.89 ± 0.23 / 4.20 ± 0.71%
$NaYF_4$:Nd/Yb/Ho	3/0.5/2%	2.47 ± 0.44 / 0.43 ± 0.15 / 1.87 ± 0.36%
$NaYF_4$:Nd/Pr	3/0.5%	3.31± 0.52 / 0.32 ± 0.22%
$NaYF_4$:Nd/Eu	3/15%	2.72± 0.44 / 15.26 ± 1.76%
$NaYF_4$:Nd/Yb/Tm	3/1/0.2%	2.86 ± 0.32 / 0.89 ± 0.17 / 0.17 ± 0.11%
$NaYF_4$:Nd/Ce	3/20%	2.33± 0.64 / 16.35 ± 2.01%

Supplementary Fig. 4 | The EDS and ICP analysis results of the as-prepared nanoparticles. a, b EDS spectra of the $NaYF_4$:Nd (3%) and Nd/Yb/Er (3/1/5%) nanoparticles. **c** ICP emission spectrometer analysis of the $NaYF_4$:Nd (3%), Nd/Yb/Er (3/1/5%), Nd/Yb/Ho (3/0.5/2%) , Nd/Pr (3/0.5%), Nd/Eu (3/15%), Nd/Yb/Tm (3/1/0.2%) and Nd/Ce (3/20%) nanoparticles. Each experiment was repeated 3 times independently with similar results.

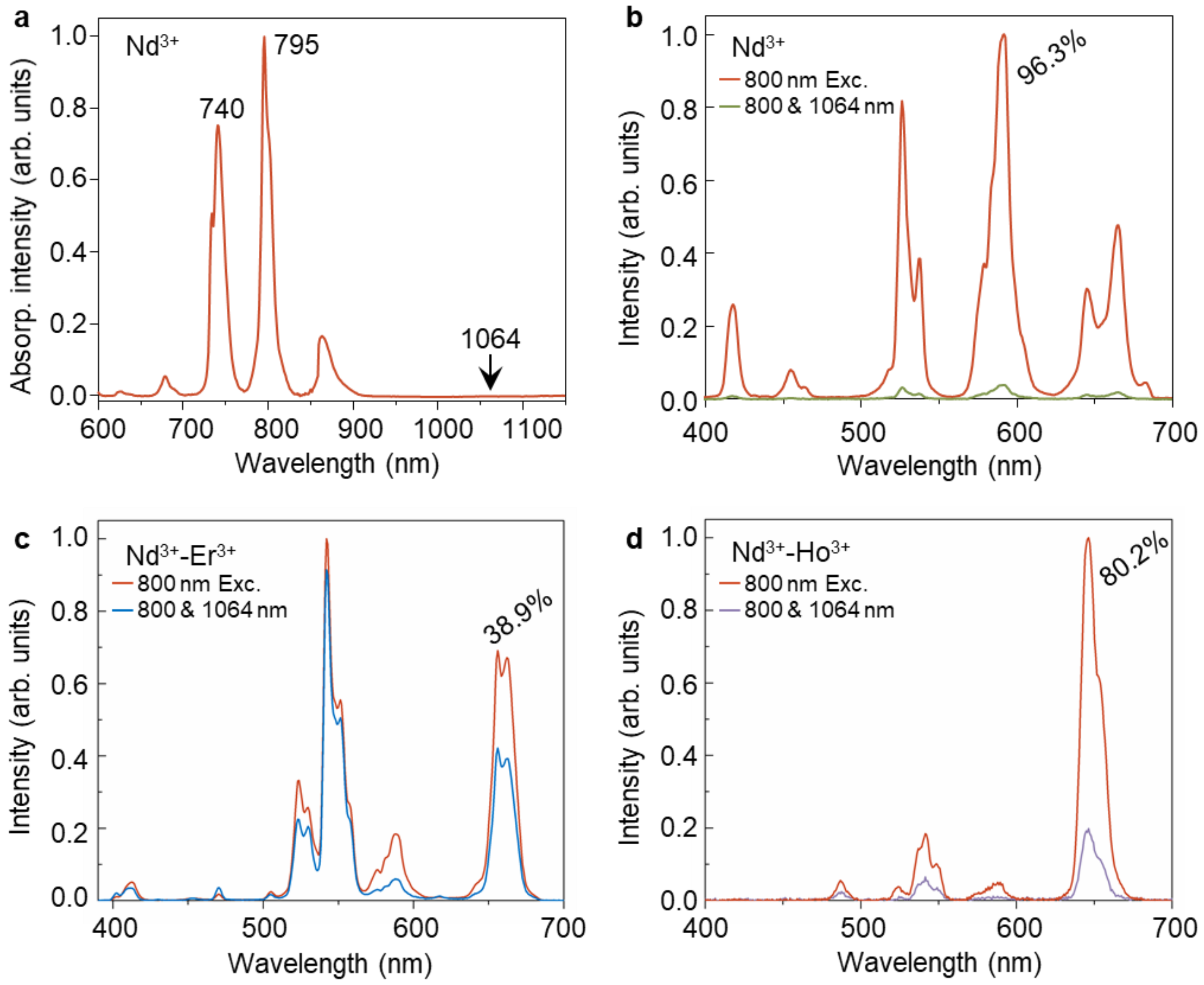

Supplementary Fig. 5 | Nd^{3+} absorption spectrum and emission inhibition under 800 nm excitation. **a** The absorption spectrum of Nd^{3+} ions, showing a strong absorption band at 740 nm, and almost no absorption at 1064 nm[1]. **b-d** Emission inhibition of $NaYF_4$: Nd (3%), Nd/Yb/Er (3/1/5%) and Nd/Yb/Ho (3/0.5/2%) nanoparticles with the co-irradiation of 800 nm and 1064 nm beam. The measured depletion efficiencies are 96.3 ± 0.3% (Nd-588 nm), 38.9 ± 1.7% (Er-655 nm), 80.2 ± 0.6% (Ho-645 nm). Uncertainties are defined as the standard deviation of n = 3 independent measurements.

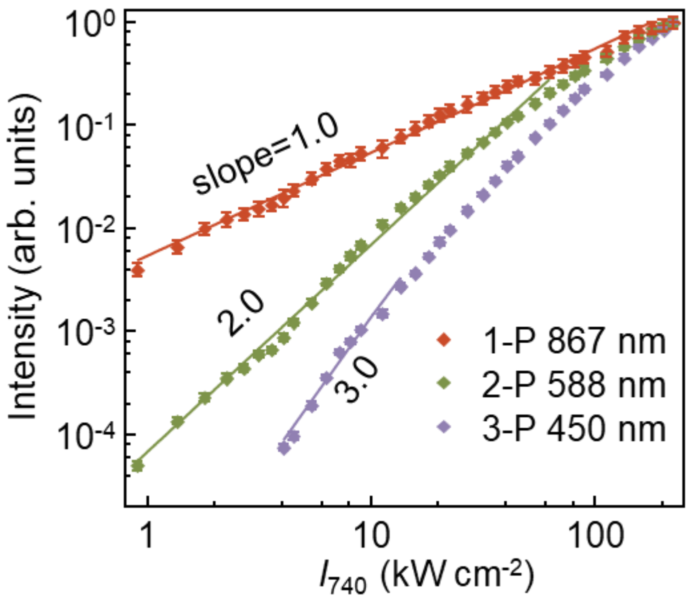

Supplementary Fig. 6 | Emission intensities from $NaYF_4$:Nd (3%) nanoparticles versus excitation intensity. The slopes denote the number of photons absorbed in the upconversion process. Data are presented as mean values +/- standard deviation (SD). Error bars are defined as the SD of n = 3 independent measurements.

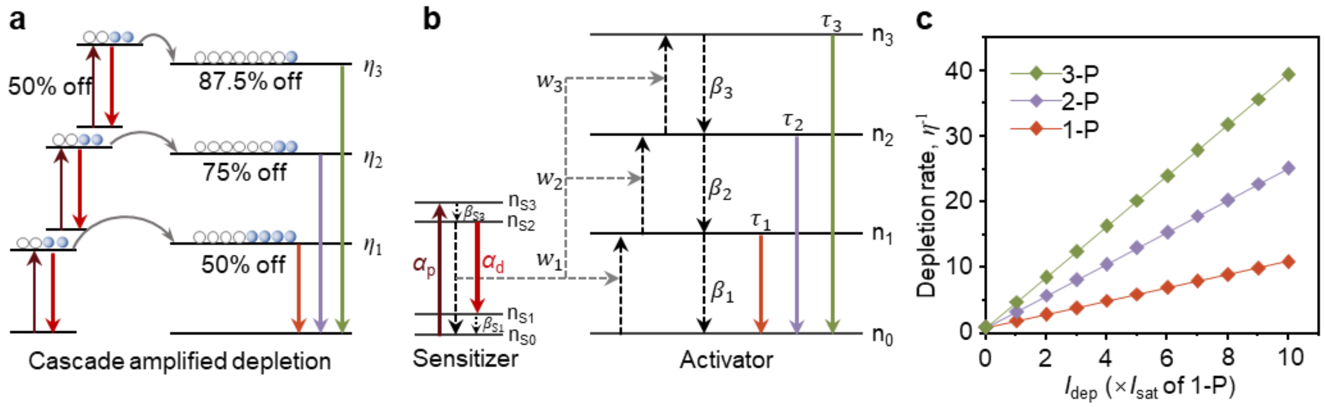

Supplementary Fig. 7 | Energy diagram of cascade amplified depletion modelling in STExD. a The depletion efficiencies of 1-, 2-, and 3-photon emissions are 50%, 75% and 87.5% with 50% depletion of sensitizer. **b** The Energy diagram of cascade amplified depletion modelling in the STExD process. **c** The calculated depletion rates ($\eta^{-1} = 1 + I/I_{sat}$) of multiphoton emissions versus depletion intensity.

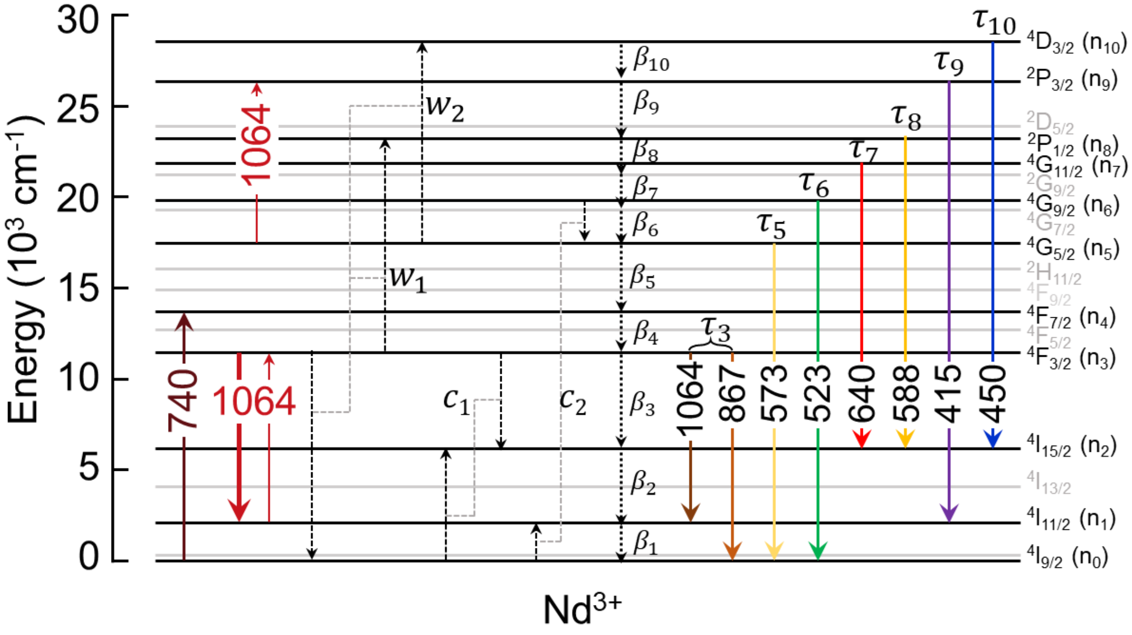

Supplementary Fig. 8 | Schematic energy diagram for the numerical simulation of the STExD mechanism with cascade amplified depletion in the $NaYF_4$:Nd system.

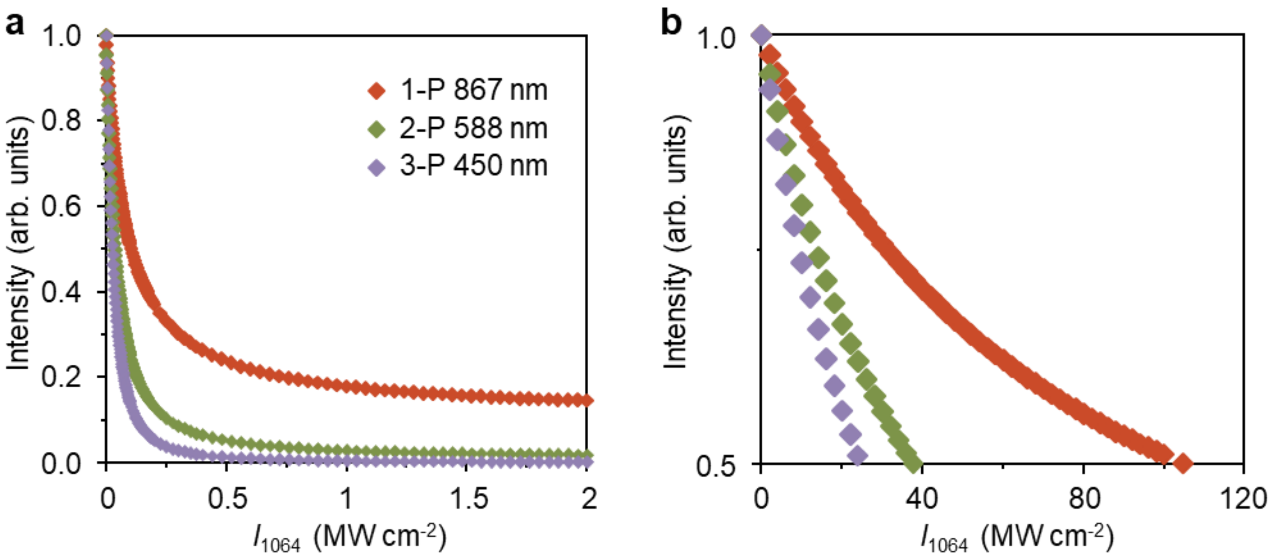

Supplementary Fig. 9 | Simulated emission intensities of $NaYF_4$:Nd (3%) nanoparticles versus depletion intensity under cascade amplified depletion effect. I_{740} = 78 kW cm^{-2}; I_{1064} = 0-2 MW cm^{-2}. The calculated depletion saturation intensities for the one-photon 867 nm, the two-photon 588 nm and the three-photon 450 nm emissions were 107.8 kW cm^{-1}, 37.9 kW cm^{-1} and 24.7 kW cm^{-1}, respectively.

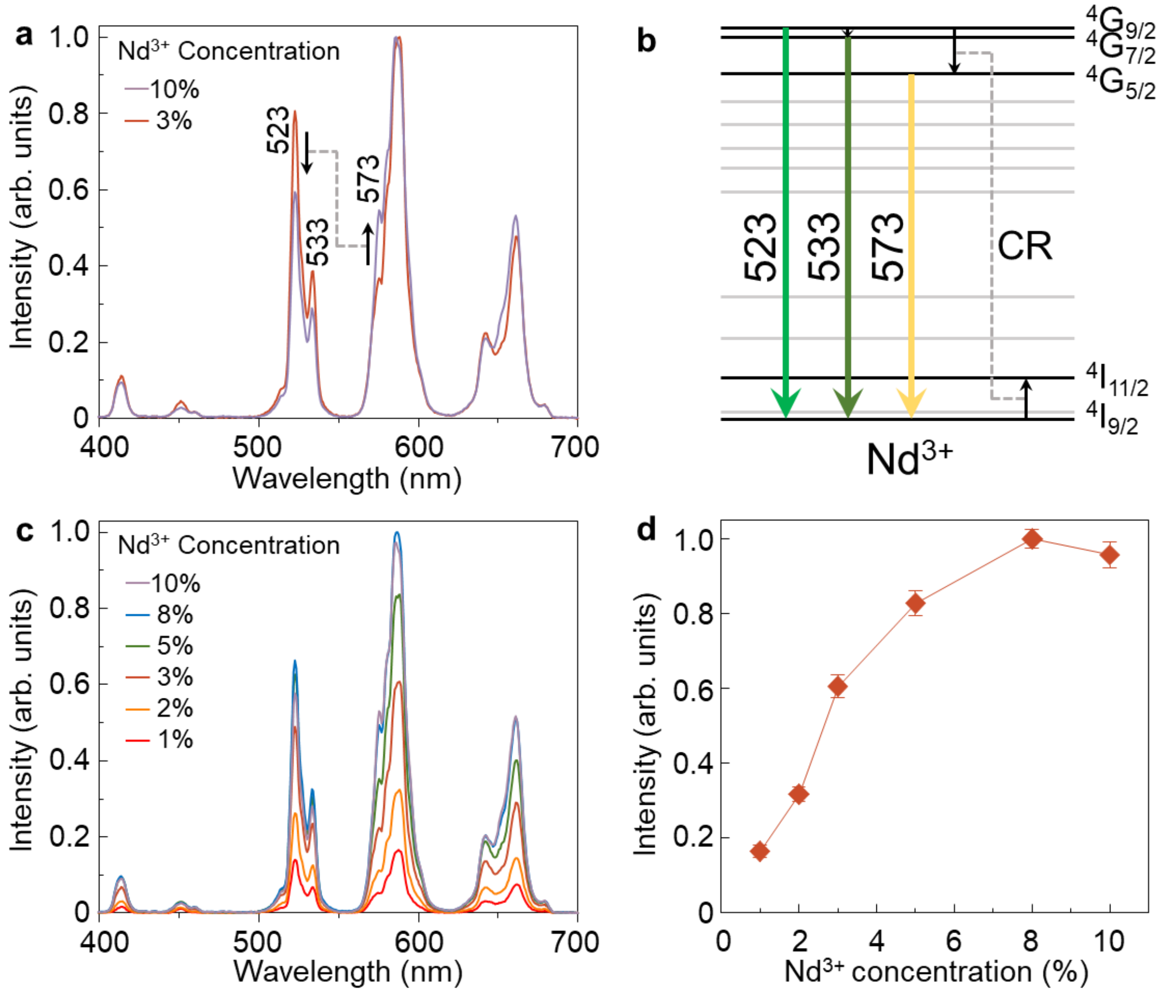

Supplementary Fig. 10 | Emission spectra and intensities versus Nd^{3+} doping concentration. a Normalized emission spectra show the weakened emission bands of 523 nm ($^4G_{9/2} \rightarrow {}^4I_{9/2}$) and 533 nm ($^4G_{7/2} \rightarrow {}^4I_{9/2}$) and the enhanced emission band of 573 nm ($^4G_{5/2} \rightarrow {}^4I_{9/2}$) in $NaYF_4$:Nd (10%) nanoparticles compared with Nd (3%). **b** The associated energy level diagram of the cross-relaxation (CR) process: $^4G_{9/2} + {}^4I_{9/2} \rightarrow {}^4G_{5/2} + {}^4I_{11/2}$. **c** Emission spectra of $NaYF_4$:Nd (x%) nanoparticles with x = 1, 2, 3, 5, 8, 10. **d** Measured emission intensities of 588 nm emission in (**c**). Data are presented as mean values +/- standard deviation (SD). Error bars are defined as the SD of n = 3 independent measurements. I_{740} = 78.3 kW cm^{-2}.

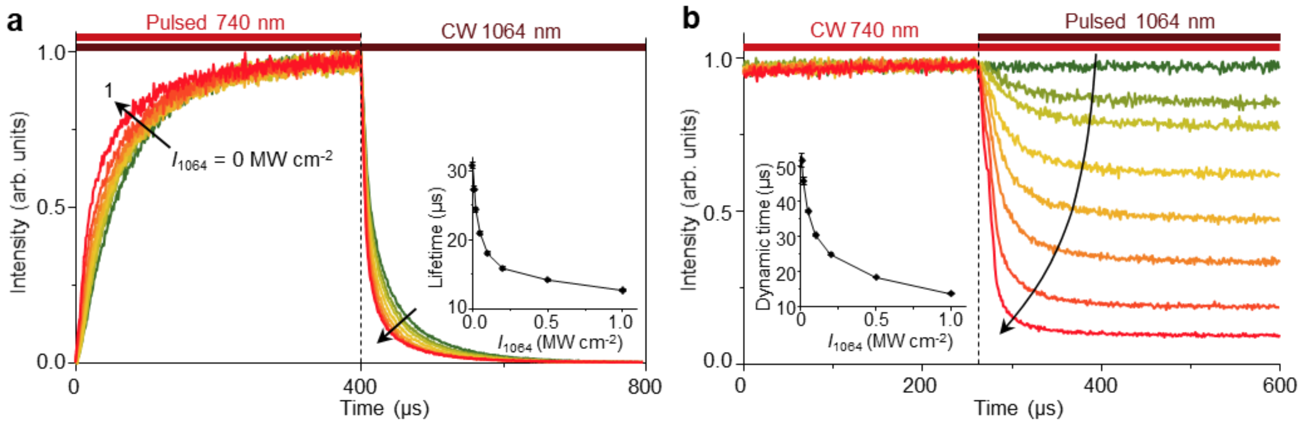

Supplementary Fig. 11 | Time-dependent kinetic processes of the $^2P_{1/2}$ state in $NaYF_4$:Nd (3%) nanoparticles with different depletion intensities. a The excitation beam was modulated with chopper (700 Hz, I_{740} = 78.3 kW cm^{-2}) and the intensity of CW depletion beam was controlled (I_{1064} = 0, 0.01, 0.02, 0.05, 0.1, 0.2, 0.5 and 1.0 MW cm^{-2}). The rising time decreased from 79.1 ± 0.5 to 46.5 ± 0.7 μs. The lifetime decreased from 30.8 ± 0.5 to 12.7± 0.2 μs. **b** The intensity of CW excitation beam was kept at I_{740} = 78.3 kW cm^{-2}, while the depletion beam was modulated with chopper (700 Hz) and the intensity was controlled (I_{1064} = 0, 0.01, 0.02, 0.05, 0.1, 0.2, 0.5 and 1.0 MW cm^{-2}). The time required to achieve steady state conditions decreased from 51.7 ± 1.9 to 13.8 ± 0.1 μs. Data are presented as mean values +/- standard deviation (SD). Error bars are defined as the SD of n = 3 independent measurements.

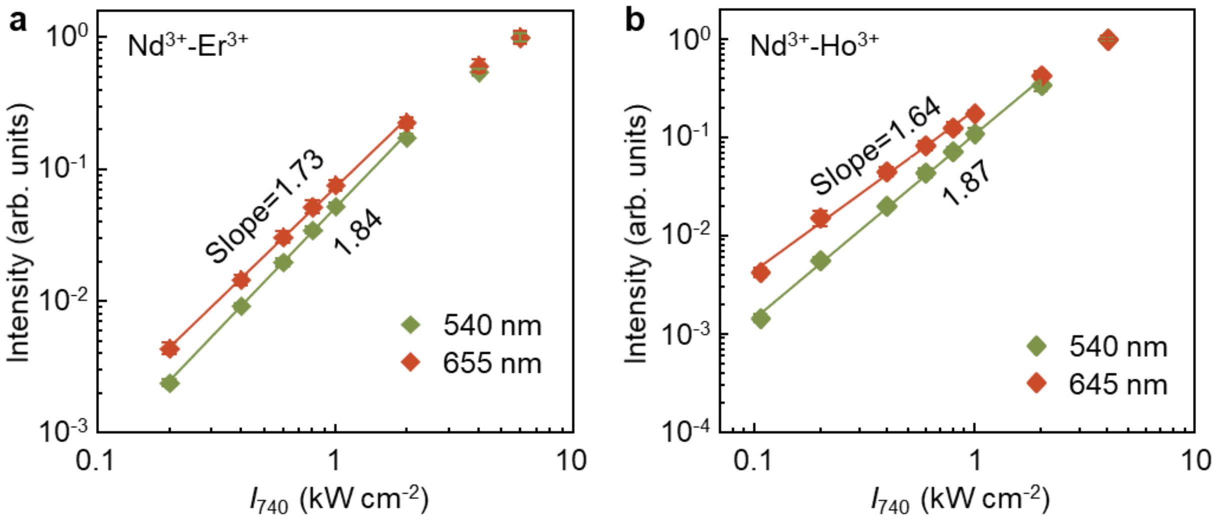

Supplementary Fig. 12 | Emission intensities of $NaYF_4$:Nd/Yb/Er (3/1/5%) and Nd/Yb/Ho (3/0.5/2%) nanoparticles versus excitation intensity. The slopes denote the number of photons absorbed in the upconversion process. Data are presented as mean values +/- standard deviation (SD). Error bars are defined as the SD of n = 3 independent measurements.

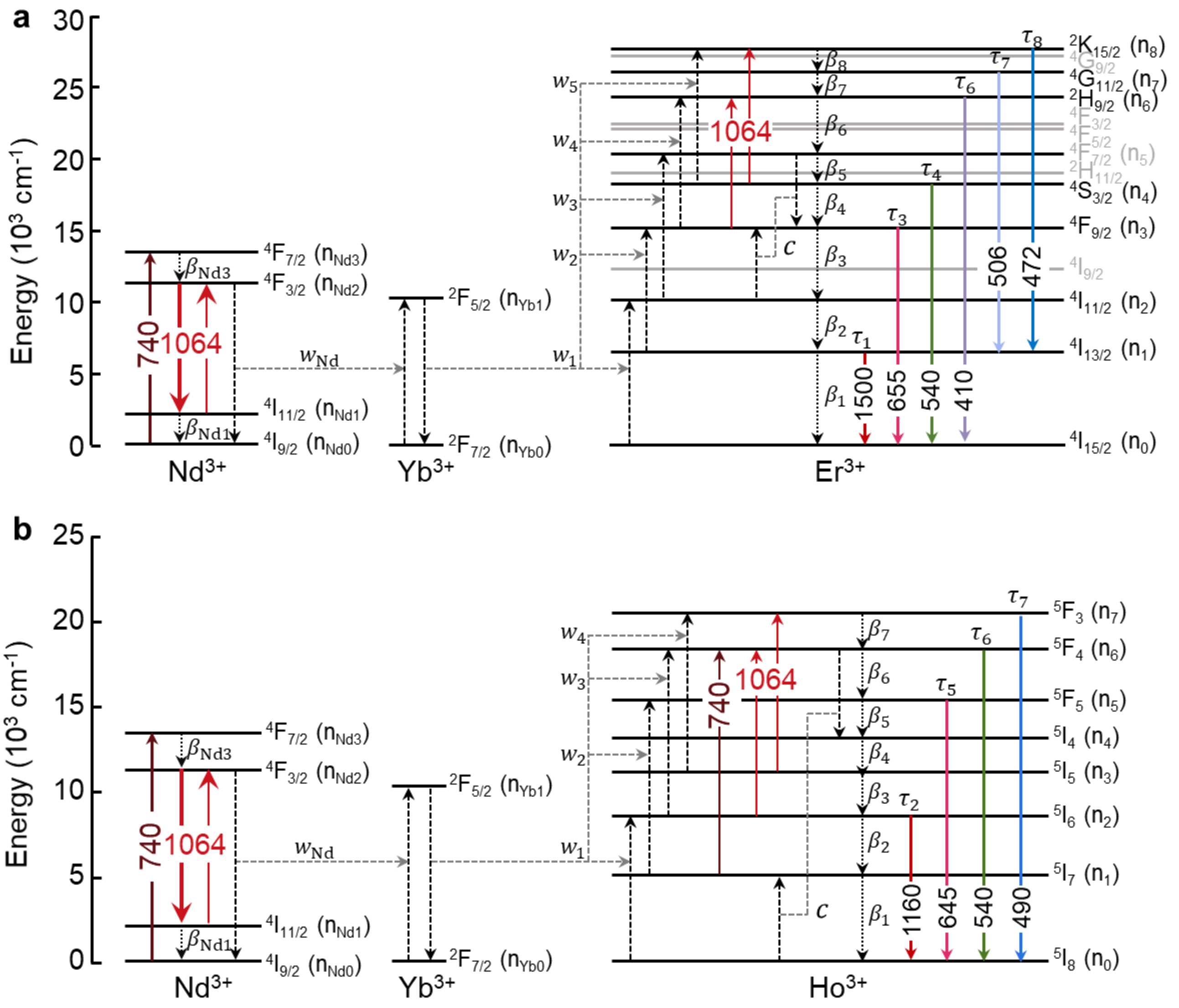

Supplementary Fig. 13 | Schematic energy diagrams for the STExD numerical simulation in (**a**) Nd/Yb/Er and (**b**) Nd/Yb/Ho system.

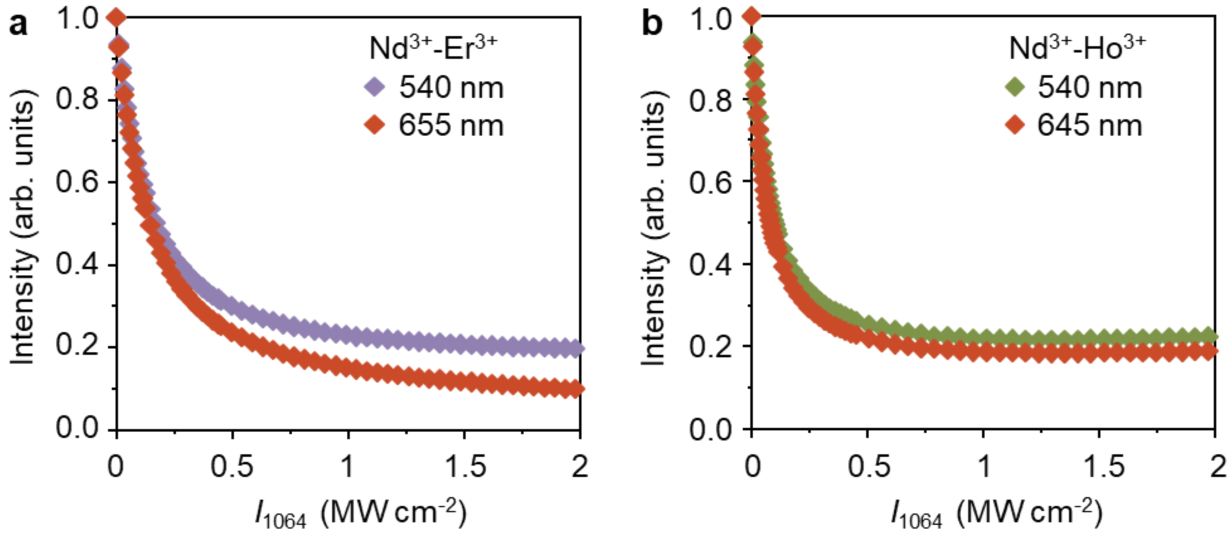

Supplementary Fig. 14 | Simulated emission intensities of $NaYF_4$:Nd/Yb/Er (3/1/5%) and $NaYF_4$:Nd/Yb/Ho (3/0.5/2%) nanoparticles versus depletion intensity. I_{740} = 78 kW cm^{-2}; I_{1064} = 0-2.25 MW cm^{-2}. **a** The calculated depletion saturation intensities of the 540 nm and 655 nm emissions from $NaYF_4$:Nd/Yb/Er (3/1/5%) are 167 kW cm^{-1} and 149 kW cm^{-1}, respectively. **b** The calculated depletion saturation intensities of the 540 nm and 645 nm emissions from $NaYF_4$: Nd/Yb/Ho (3/0.5/2%) are 98 kW cm^{-1} and 81 kW cm^{-1}, respectively.

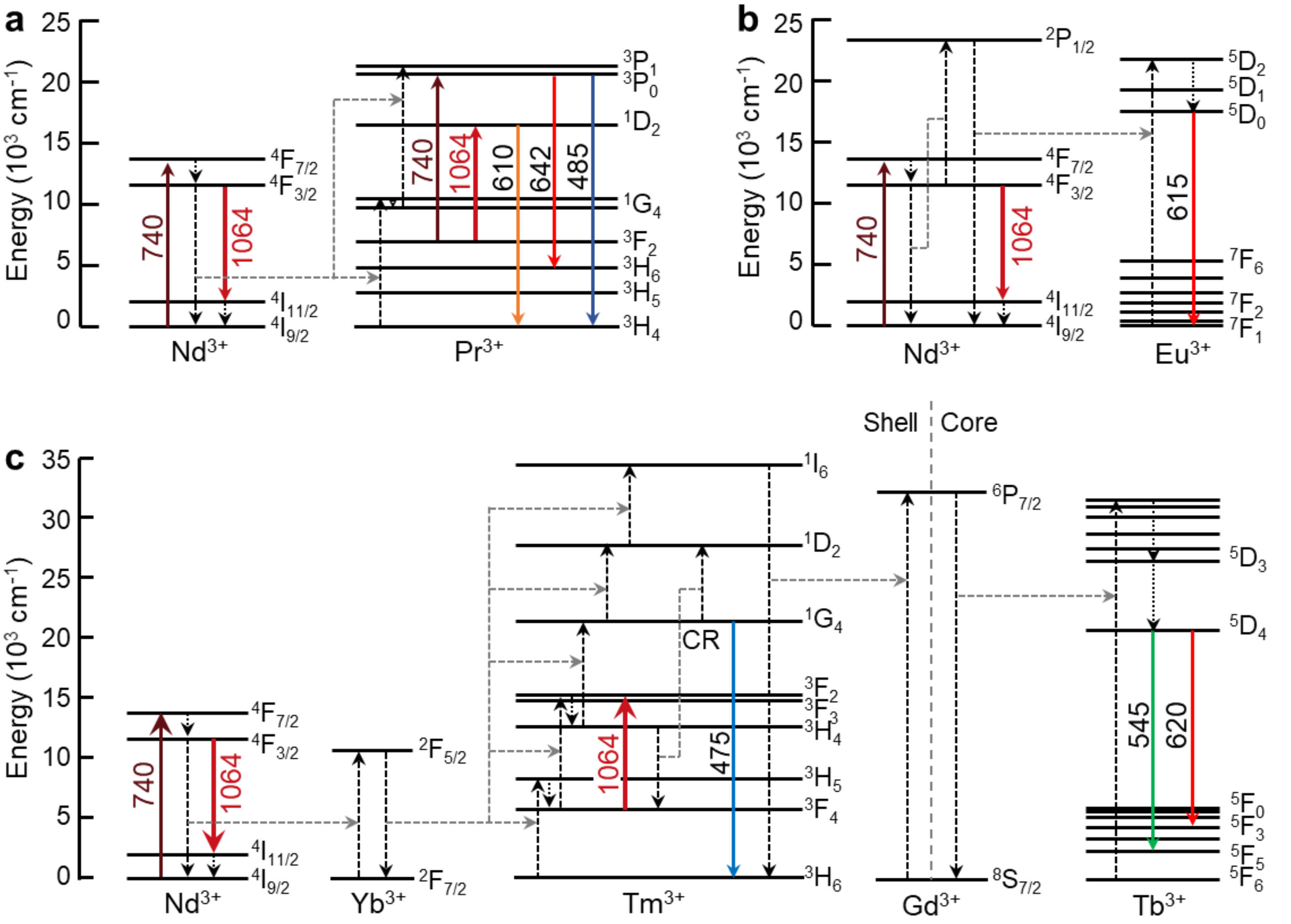

Supplementary Fig. 15 | Schematic energy diagrams of **(a)** the Nd/Pr, **(b)** the Nd/Eu, **(c)** the Nd/Yb/Tm and Gd/Tb@Gd/Nd/Yb/Tm systems.

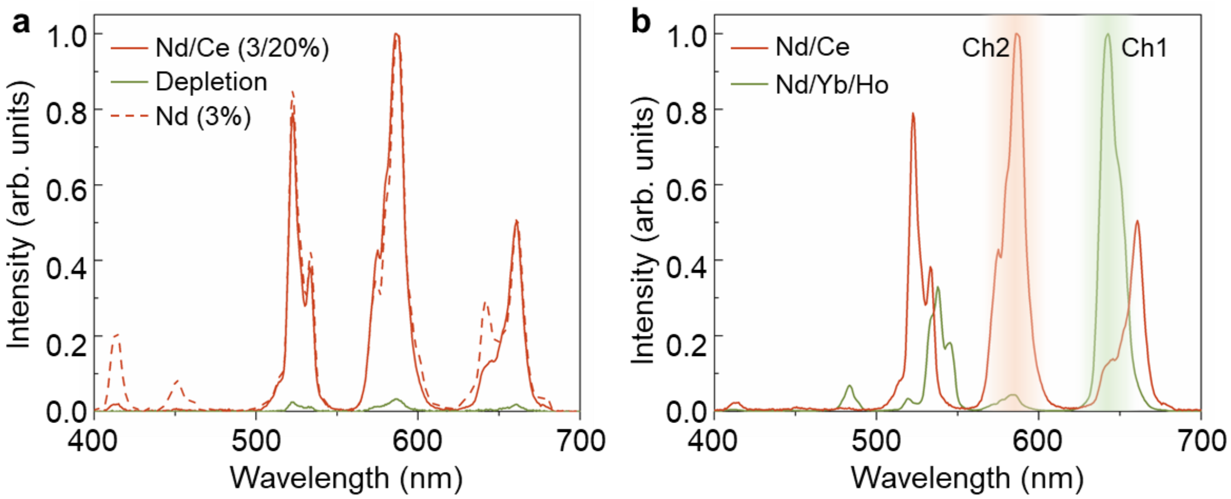

Supplementary Fig. 16 | Emission spectra from nanoprobes for two-color STExD imaging. a Full-spectrum emission inhibition of $NaYF_4$:Nd/Ce (3/20%) nanoparticles under 740 nm and 1064 nm co-irradiation (I_{740} = 78.3 kW cm^{-2}; I_{1064} = 2.5 MW cm^{-2}). **b** Merged emission spectra of $NaYF_4$:Nd/Ce (3/20%) and $NaYF_4$:Nd/Yb/Ho (3/0.5/2%) nanoparticles. The spectral range for fluorescence detection of channel 1 (Ch1) and channel 2 (Ch2) are highlighted with green and brown.

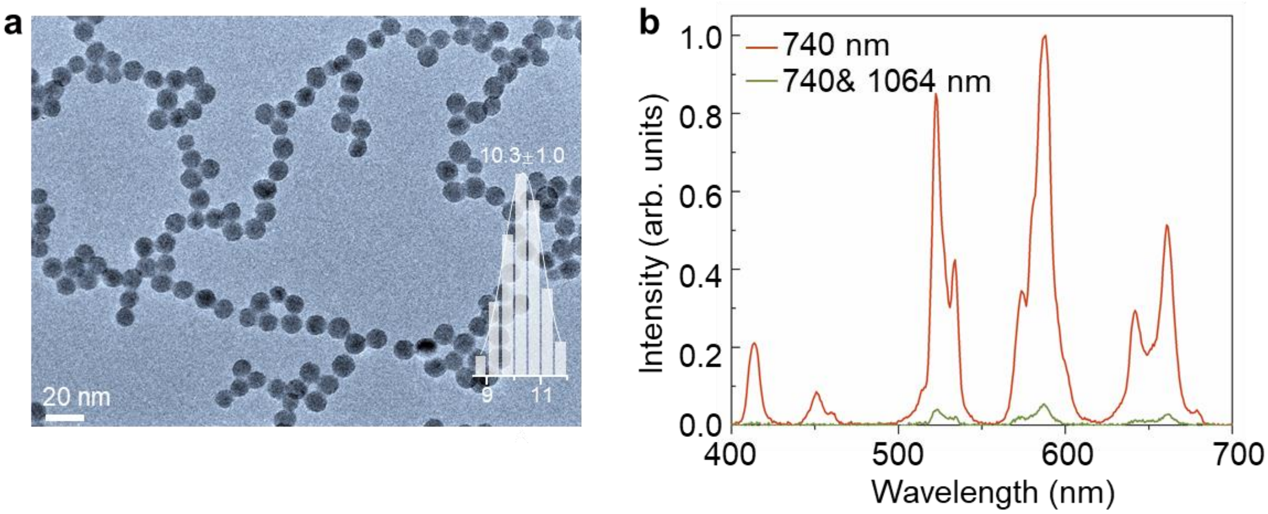

Supplementary Fig. 17 | TEM image and spectrum of the phalloidin modified $NaYF_4$:Nd (3%) nanoparticles. a TEM image of phalloidin modified $NaYF_4$:Nd (3%) nanoparticles, average size: 10.3 ± 1.0 nm in diameter. The measurement was repeated 3 times independently with similar results. **b** Emission inhibition of phalloidin modified $NaYF_4$:Nd (3%) nanoparticles under 740 nm and 1064 nm co-irradiation (I_{740} = 78.3 kW cm^{-2}; I_{1064} = 2.5 MW cm^{-2}). This result shows that the modification procedures would not affect the dispersibility and optical depletion performance of the nanoparticles.

Supplementary Table 1. Comparison of the theoretical saturation intensity of different emitters.

**Energy state and wavelength λ**	**Stimulated emission cross-section σ**	**Radiative decay lifetime τ**	**Depletion saturation intensity I_{sat} [$\propto(\sigma\times\tau\times\lambda)^{-1}$]**
Er^{3+}: $^4I_{13/2}$@1550 nm[a]	5.56×10^{-21} cm^2	9.01 ms	$I_{sat} \propto 1.29 \times 10^{23}$ cm^{-3} ms^{-1}
Ho^{3+}: $^5I_7$@1950 nm[b]	4.71×10^{-21} cm^2	12.5 ms	$I_{sat} \propto 8.71\times10^{22}$ cm^{-3} ms^{-1}
Yb^{3+}: $^2F_{5/2}$@1050 nm[c]	2.3×10^{-20} cm^2	1.5 ms	$I_{sat} \propto 2.76\times10^{23}$ cm^{-3} ms^{-1}
Nd^{3+}: $^4F_{3/2}$@1064 nm[d]	1.2×10^{-19} cm^2	200 μs	$I_{sat} \propto 3.92\times10^{23}$ cm^{-3} ms^{-1}
Dyes JA 26@ 532 nm[e]	1.2×10^{-16} cm^2	4.0 ns	$I_{sat} \propto 3.92\times10^{25}$ cm^{-3} ms^{-1}

[a] *J. Lumin.* **189**, 84–90 (2017)[2]; *Nat. Commun.* **8**, 1–11 (2017)[3]

[b] *Opt. Mater.* **75**, 695–698 (2018)[4]; *J. Alloys Compd.* **380**, 156–158 (2004)[5]

[c] *J. Opt. Soc. Am. B* **20**, 1975 (2003)[6]

[d] *Mater. Res. Bull.* **60**, 838–842 (2014)[7]; *J. Opt. Soc. Am. B* **3**, 102 (1986)[8];

[e] *Angew. Chem. Int. Ed.* **43**, 6646–6649 (2004)[9]

Supplementary Table 2. The list of the as-synthesized samples studied in this work.

Samples	No. of Figure	Measurements
$NaYF_4$:Nd (3%) (10.6 ± 1.0 nm)	Fig. 2b, 2c, 2e; Supplementary Fig. 3a, 4a, 4c, 5a, 5b, 6, 10, 11	Emission inhibition measurements and STExD mechanism investigation
$NaYF_4$:Nd (x%, x = 1, 2, 3, 4, 5, 8, 10) (about 10 nm in average)	Supplementary Fig. 10	
$NaYF_4$:Nd/Yb/Er (3/1/5%) (8.4 ± 1.0 nm)	Fig. 3b, 3c; Supplementary Fig. 3b, 4b, 4c, 5c, 12a	
$NaYF_4$:Nd/Yb/Ho (3/0.5/2%) (9.1 ± 1.0 nm)	Fig. 3c, 3d; Supplementary Fig. 3c, 4c, 5d, 12b	
$NaYF_4$:Nd/Yb/Tm (3/1/0.2%) (8.9 ± 0.9 nm)	Fig. 3g; Supplementary Fig. 4c	
$NaYF_4$:Nd/Pr (3/0.5%) (9.6 ± 0.9 nm)	Fig. 3e; Supplementary Fig. 4c	
$NaYF_4$:Gd/Tb (50/15%)@$NaYF_4$:Gd/Nd/Yb/Tm (50/3/5/1%) (18.4 ± 1.4 nm)	Fig. 3h	
$NaYF_4$:Nd/Eu (3/10%) (10.4 ± 1.1 nm)	Fig. 3f; Supplementary Fig. 4c	
$NaYF_4$:Nd (3%) (16.7 ± 0.9 nm)	Fig. 4b, 4c; Supplementary Fig. 16a	STExD multi-imaging and two-color imaging
$NaYF_4$:Nd/Yb/Er (3/1/5%) (20.5 ± 1.0 nm)	Fig. 4d	
$NaYF_4$:Nd/Yb/Ho (3/0.5/2%) (21.3 ± 0.9 nm)	Fig. 4e, 4f; Supplementary Fig. 16b	
$NaYF_4$:Nd/Ce (3/20%) (17.9 ± 1.2 nm)	Fig. 4f; Supplementary Fig. 16	
$NaYF_4$:Nd (3%) (10.3 ± 1.0 nm) (phalloidin modified)	Fig. 5; Supplementary Fig. 17	Immunolabeling and super-resolution imaging of Hela cells

Supplementary Table 3. Measured depletion efficiencies of different emissions of different Nd-sensitizing nanoparticles.

Sample	λ_{em} and transition	Depletion efficiency
$NaYF_4$:Nd (3%)	900 nm ($^4F_{3/2} \rightarrow {}^4I_{9/2}$)	87.1 ± 0.6%
	867 nm ($^4F_{3/2} \rightarrow {}^4I_{9/2}$)	87.7 ± 0.4%
	660 nm ($^2G_{9/2} \rightarrow {}^4I_{15/2}$)	97.0 ± 0.3%
	588 nm ($^2P_{1/2} \rightarrow {}^4I_{15/2}$)	96.4 ± 0.4%
	523 nm ($^4G_{9/2} \rightarrow {}^4I_{9/2}$)	96.1 ± 0.4%
	450 nm ($^4D_{3/2} \rightarrow {}^4I_{15/2}$)	99.3 ± 0.3%
	415 nm ($^4P_{3/2} \rightarrow {}^4I_{11/2}$)	97.8 ± 0.5%
$NaYF_4$:Nd/Yb/Er (3/1/5/%)	655 nm ($^4F_{9/2} \rightarrow {}^4I_{15/2}$)	90.3 ± 0.4%
	540 nm ($^4S_{3/2} \rightarrow {}^4I_{15/2}$)	80.7 ± 0.4%
	525 nm ($^2H_{11/2} \rightarrow {}^4I_{15/2}$)	74.3 ± 0.8%
	506 nm ($^4G_{11/2} \rightarrow {}^4I_{13/2}$)	78.9 ± 0.6%
	472 nm ($^2K_{15/2} \rightarrow {}^4I_{13/2}$)	46.9 ± 0.8%
	410 nm ($^2H_{9/2} \rightarrow {}^4I_{15/2}$)	79.0 ± 0.7%
$NaYF_4$:Nd/Yb/Ho (3/0.5/2%)	645 nm ($^5F_5 \rightarrow {}^5I_8$)	82.2 ± 0.3%
	540 nm ($^5F_4 \rightarrow {}^5I_8$)	77.3 ± 0.6%
	488 nm ($^5F_3 \rightarrow {}^5I_8$)	70.0 ± 0.4%
$NaYF_4$:Nd/Pr (3/0.5%)	642 nm ($^3P_0 \rightarrow {}^3H_6$)	46.2 ± 0.6%
	610 nm ($^1D_2 \rightarrow {}^3H_4$)	35.5 ± 0.7%
	485 nm ($^3P_0 \rightarrow {}^3H_4$)	35.0 ± 0.9%
$NaYF_4$:Nd/Eu (3/15%)	615 nm ($^5D_0 \rightarrow {}^7F_1$)	88.1 ± 1.7%
$NaYF_4$:Nd/Yb/Tm (3/1/0.2%)	475 nm ($^1G_4 \rightarrow {}^3H_6$)	55.1 ± 1.3%
$NaYF_4$:Gd/Tb@Gd/Nd/Yb/Tm (50/15@50/3/5/1%)	620 nm ($^5D_4 \rightarrow {}^5F_3$)	48.8 ± 0.9%
	545 nm ($^5D_4 \rightarrow {}^5F_5$)	58.1 ± 0.7%

Supplementary Table 4. The values of parameters used in the simulation for $NaYF_4$:Nd system.

τ_3 (μs)	τ_5 (μs)	τ_6 (μs)	τ_7 (μs)	τ_8 (μs)	τ_9 (μs)	τ_{10} (μs)
200[ad]	50[b]	50	50[b]	50[b]	40[c]	30
β_1 (s^{-1})	β_2 (s^{-1})	β_3 (s^{-1})	β_4 (s^{-1})	β_5 (s^{-1})	β_6 (s^{-1})	β_7 (s^{-1})
5×10^{5}[d]	5×10^{4}[d]	2×10^{3}	3×10^{4}[d]	3×10^{4}	2×10^{4}	3×10^{4}
β_8 (s^{-1})	β_9 (s^{-1})	β_{10} (s^{-1})	w_1 (cm^3 s^{-1})	w_2 (cm^3 s^{-1})	c_1 (cm^3 s^{-1})	c_2 (cm^3 s^{-1})
1.3×10^{4}	1×10^{4}	1.2×10^{4}	1.2×10^{-16}	5×10^{-17}	1.6×10^{-16}	5×10^{-17}
σ_0 (cm^2)	σ_{13} (cm^2)	σ_{31} (cm^2)	σ_{59} (cm^2)	ρ_1 (kW cm^{-2})	ρ_2 (kW cm^{-2})	
3.6×10^{-20}[e]	1.2×10^{-19}[e]	1.2×10^{-19}[e]	3×10^{-23}	78	0 ~ 2000	
b_{30}	b_{31}					
0.4[f]	0.6[f]					

[a] *J. Phys. Chem. C* **118**, 13087–13098 (2014)[10]

[b] *Opt. Commun.* **147**, 203–211 (1998)[11]

[c] *J. Lumin.* **45**, 346–350 (1990)[12]

[d] *Nanoscale Horiz.* **4**, 881–889 (2019)[13],

[e] *Mater. Res. Bull.* **60**, 838–842 (2014)[7]; *J. Opt. Soc. Am. B* **3**, 102 (1986)[8]

[f] *Appl. Opt.* **41**, 7052 (2002)[14]

Supplementary Table 5. The values of parameters used in the simulation for $NaYF_4$:Nd/Yb/Er system.

τ_1 (μs)	τ_3 (μs)	τ_4 (μs)	τ_6 (μs)	τ_7 (μs)	τ_8 (μs)
500[a]	200[a]	140[b]	90[c]	150	70
β_1 (s^{-1})	β_2 (s^{-1})	β_3 (s^{-1})	β_4 (s^{-1})	β_5 (s^{-1})	β_6 (s^{-1})
8×10^3	3×10^4	4×10^4	5×10^4	8×10^4	6×10^4
β_7 (s^{-1})	β_8 (s^{-1})	β_{Nd1} (s^{-1})	β_{Nd3} (s^{-1})	w_1 (cm^3 s^{-1})	w_2 (cm^3 s^{-1})
4×10^4	4×10^4	5×10^5	5×10^4	1.4×10^{-18}[d]	1×10^{-18}
w_3 (cm^3 s^{-1})	w_4 (cm^3 s^{-1})	w_5 (cm^3 s^{-1})	w_{Nd} (cm^3 s^{-1})	c (cm^3 s^{-1})	σ_{Nd0} (cm^2)
1.4×10^{-18}	1×10^{-18}	5×10^{-19}	5.5×10^{-17}	8×10^{-17}	3.6×10^{-20}
σ_{Nd12} (cm^2)	σ_{Nd21} (cm^2)	σ_{36} (cm^2)	σ_{48} (cm^2)	ρ_1 (kW cm^{-2})	ρ_2 (kW cm^{-2})
1.2×10^{-19}	1.2×10^{-19}	2×10^{-21}	2.5×10^{-21}	78	0 ~ 2000

[a] *Opt. Mater.* **6**, 267–274 (1996)[15]

[b] *J. Lumin.* **189**, 84–90 (2017)[2]

[c] *Phys. Rev. B* **56**, 1800–1808 (1997)[16]

[d] *J. Opt. Soc. Am. B* **17**, 833 (2000)[17]

Supplementary Table 6. The values of parameters used in the simulation for $NaYF_4$:Nd/Yb/Ho system.

τ_2 (μs)	τ_5 (μs)	τ_6 (μs)	τ_7 (μs)	β_1 (s^{-1})	β_2 (s^{-1})
500[a]	200[b]	200[c]	120	1×10^4	3×10^4
β_3 (s^{-1})	β_4 (s^{-1})	β_5 (s^{-1})	β_6 (s^{-1})	β_7 (s^{-1})	β_{Nd1} (s^{-1})
3×10^4	5×10^4	5×10^4	3×10^4	5×10^4	5×10^5
β_{Nd3} (s^{-1})	w_{Nd} (cm^3 s^{-1})	w_1 (cm^3 s^{-1})	w_2 (cm^3 s^{-1})	w_3 (cm^3 s^{-1})	w_4 (cm^3 s^{-1})
5×10^4	3×10^{-17}	1×10^{-17}	2×10^{-17}	1.2×10^{-17}	6×10^{-18}
c (cm^3 s^{-1})	σ_{Nd0} (cm^2)	σ_{Nd12} (cm^2)	σ_{Nd21} (cm^2)	σ_{16} (cm^2)	σ_{26} (cm^2)
5×10^{-18}	3.6×10^{-20}	1.2×10^{-19}	1.2×10^{-19}	1.2×10^{-20}	6×10^{-22}
σ_{37} (cm^2)	ρ_1 (kW cm^{-2})	ρ_2 (kW cm^{-2})			
2.2×10^{-23}	78	0 ~ 2000			

[a] *J. Alloys Compd.* **380**, 362–367 (2004)[18]

[b] *Phys. B Condens. Matter* **406**, 1248–1252 (2011)[19]

[c] *J. Appl. Phys.* **93**, 9460–9465 (2003)[20]

Supplementary References:

1 Liao, J., Jin, D., Chen, C., Li, Y. & Zhou, J. Helix Shape Power-Dependent Properties of Single Upconversion Nanoparticles. *J. Phys. Chem. Lett.* **11**, 2883-2890 (2020).

2 Villanueva-Delgado, P., Biner, D. & Krämer, K. W. Judd–Ofelt analysis of β-$NaGdF_4$: Yb^{3+}, Tm^{3+} and β-$NaGdF_4$:Er^{3+} single crystals. *J. Lumin.* **189**, 84-90 (2017).

3 Zhan, Q. *et al.* Achieving high-efficiency emission depletion nanoscopy by employing cross relaxation in upconversion nanoparticles. *Nat. Commun.* **8**, 1058 (2017).

4 Cao, W. *et al.* 2.0 µm emission of Ho^{3+} doped germanosilicate glass sensitized by non-rare-earth ion Bi: A new choice for 2.0 µm laser. *Opt. Mater.* **75**, 695-698 (2018).

5 Kowalska, M., Klocek, G., Piramidowicz, R. & Malinowski, M. Ultra-violet emission in Ho:ZBLAN fiber. *J. Alloys Compd.* **380**, 156-158 (2004).

6 Dong, J., Bass, M., Mao, Y., Deng, P. & Gan, F. Dependence of the Yb^{3+} emission cross section and lifetime on temperature and concentration in yttrium aluminum garnet. *J. Opt. Soc. Am. B* **20**, 1975-1979 (2003).

7 Singh, G., Tiwari, V. S. & Gupta, P. K. Spectroscopic analysis on the basis Judd–Ofelt theory of Nd^{3+} in $(Y_{0.985}Nd_{0.015})_2O_3$: A transparent laser-host ceramic. *Mater.Res. Bull.* **60**, 838-842 (2014).

8 Krupke, W. F., Shinn, M. D., Marion, J. E., Caird, J. A. & Stokowski, S. E. Spectroscopic, optical, and thermomechanical properties of neodymium- and chromium-doped gadolinium scandium gallium garnet. *J. Opt. Soc. Am. B* **3**, 102-114 (1986).

9 Kastrup, L. & Hell, S. W. Absolute Optical Cross Section of Individual Fluorescent Molecules. *Angew.Chem. Int. Ed.* **43**, 6646-6649 (2004).

10 Skrzypczak, U., Pfau, C., Seifert, G. & Schweizer, S. Comprehensive Rate Equation Analysis of Upconversion Luminescence Enhancement Due to $BaCl_2$ Nanocrystals in Neodymium-Doped Fluorozirconate-Based Glass Ceramics. *J. Phys. Chem. C* **118**, 13087-13098 (2014).

11 Pollnau, M., Hardman, P. J., Clarkson, W. A. & Hanna, D. C. Upconversion, lifetime quenching, and ground-state bleaching in Nd^{3+}:$LiYF_4$. *Opt. Commun.* **147**, 203-211 (1998).

12 Lenth, W. & Macfarlane, R. M. Excitation mechanisms for upconversion lasers. *J. Lumin.* **45**,

346-350 (1990).

13 Bednarkiewicz, A., Chan, E. M., Kotulska, A., Marciniak, L. & Prorok, K. Photon avalanche in lanthanide doped nanoparticles for biomedical applications: super-resolution imaging. *Nanoscale Horiz.* **4**, 881-889 (2019).

14 Rapaport, A., Zhao, S., Xiao, G., Howard, A. & Bass, M. Temperature dependence of the 1.06-μm stimulated emission cross section of neodymium in YAG and in GSGG. *Appl. Opt.* **41**, 7052-7057 (2002).

15 Simondi-Teisseire, B., Viana, B., Vivien, D. & Lejus, A. M. Yb^{3+} to Er^{3+} energy transfer and rate-equations formalism in the eye safe laser material Yb:Er:$Ca_2Al_2SiO_7$. *Opt. Mater.* **6**, 267-274 (1996).

16 Riedener, T., Egger, P., Hulliger, J. & Güdel, H. U. Upconversion mechanisms in Er^{3+}-doped Ba_2YCl_7. *Phys. Rev. B* **56**, 1800-1808 (1997).

17 Hwang, B.-C. *et al.* Cooperative upconversion and energy transfer of new high Er^{3+}- and Yb^{3+}–Er^{3+}-doped phosphate glasses. *J Opt Soc Am B* **17**, 833-839 (2000).

18 Dereń, P. J. & Krupa, J. C. Spectroscopic properties of $LaAlO_3$ doped with Ho^{3+}. *J. Alloys Compd.* **380**, 362-367 (2004).

19 Chen, X. P., Zhang, W. J. & Zhang, Q. Y. Towards efficient upconversion and downconversion of $NaYF_4$:Ho^{3+},Yb^{3+} phosphors. *Physica B Condens. Matter* **406**, 1248-1252 (2011).

20 Boyer, J. C., Vetrone, F., Capobianco, J. A., Speghini, A. & Bettinelli, M. Optical transitions and upconversion properties of Ho^{3+} doped ZnO−TeO_2 glass. *J. Appl. Phys.* **93**, 9460-9465 (2003).
